# Spectrum of Preneoplastic and Neoplastic lesions of intestine in a Tertiary Care Hospital of Karachi, Pakistan

**DOI:** 10.12669/pjms.36.2.687

**Published:** 2020

**Authors:** Asma Shabbir, Muhammad Asif Qureshi, Saadia Akram, Talat Mirza

**Affiliations:** 1 Asma Shabbir, Assistant Professor, Department of Pathology, Sindh Medical College, Jinnah Sindh Medical University, Karachi, Pakistan; 2 Muhammad Asif Qureshi, Associate Professor, Dow International Medical College, Dow University of Health Sciences, Karachi, Pakistan. Sindh Medical College, Jinnah Sindh Medical University, Karachi, Pakistan; 3 Prof. Saadia Akram, Sindh Medical College, Jinnah Sindh Medical University, Karachi, Pakistan; 4 Prof. Talat Mirza, Department of Pathology & Head of Histopathology, Dean of Researc Department, Dr. Ziauddin Hospital & University, Karachi, Pakistan

**Keywords:** Squamous cell carcinoma, Adenocarcinoma, Adenoma, Preneoplastic, Neoplastic

## Abstract

**Objectives:**

To present 7 years data mentioning the spectrum of preneoplastic & neoplastic cases of intestine received at Dow Diagnostic Research and Reference Laboratory.

**Methods:**

All the cases of preneoplastic & neoplastic lesions of intestine received during 2009 – 2015 were reviewed. The data obtained were subjected to descriptive statistical analysis using SPSS version 22. Furthermore, the association of diagnosis was seen with various other variables including age, gender & site of the lesion. A p-value of < 0.05 was considered as significant.

**Results:**

The total samples were 486, out of which 33 cases were of premalignant and 453 were of malignant lesions. Out of total 33 cases of premalignant lesions of intestine, it consisted adenomatous polyp = 39.4% (n=13), dysplasia = 36.4% (n=12) and adenoma = 24.2% (n=8). From the total of 453 cases diagnosed as malignant lesions; adenocarcinoma as Grade-I were 14.2% (n=64), Grade-II were 7.6% (n=260) and Grade-III were 22% (n=99). Squamous cell carcinoma Grade-I were 0.4% (n=2), Grade-II 1.6% (n=7) and Grade-III 0.9% (n=4). 2.4% (n=11) cases were of metastatic adoncarcinoma, 0.9% (n=4) were diagnosed as neuroendocrine tumors and 0.4% (n=2) as lymphoma. A significant association was seen between site of the tumor and diagnosis, rectum was the commonest site for adenocarcinomas (p=0.001). Moderately differentiated adenocarcinoma was predominantly present in young age (p=0.001).

**Conclusion:**

Colorectal carcinoma is on rise in Pakistan, predominantly in young males, and rectum being the commonest site. In our study, all the lesions showed male predominance with adenomatous polyp as the commonest premalignant lesion & Grade-II adenocarcinoma the most common malignancy of intestine.

## INTRODUCTION

Cancer is one of the leading causes of high mortality worldwide. The Union for International Cancer Control (UICC) & International Agency for Research on Cancer (IARC) quotes that the mortality rate due to cancer has increased to 9.6 million across the globe.^1^ There is no existent national database in Pakistan from where national level statistics can be referred. Several local hospitals are operating in the country & maintaining their records but they need to contribute towards the national statistics. Shaukhat Khanum Memorial Cancer and Research Centre (SKMCRC) & Karachi Cancer Registry (KCR) are the successful models of hospital based cancer registries. Although Punjab Cancer Registry (PCR) has provided cancer statistics for Pakistan which has been reported in Globocan 2012, it does not signify the statistics for the whole country & the data has been categorized as category E (regional data rates) by Globocan 2012.^2^ Therefore, there is a desperate need to establish a national level registry in Pakistan.

Although several hospitals in the country do have their own records but we at Dow University of Health Sciences, is the largest government sector hospital in Karachi. In our setup we experienced that gastrointestinal pathology is one of the commonest components of our practice. Despite new researches & treatment modalities, gastrointestinal cancers remain an epidemiological health problem globally. According to KCR, which is the population based cancer registry in Pakistan recognized by World Health Organization (International Agency for Research on Cancer), the prevalence of gastrointestinal cancer during 2000-2008 is 6.9%.^3^

In this study, our objective was to present seven years data mentioning the spectrum of preneoplastic & neoplastic cases of intestine received at DDRRL. The data would help us to know the disease burden in our region. Furthermore, it would also add up to the research platform and will contribute to the establishment of national registry in future.

## METHODS

Cross sectional prospective study was conducted at Dow Diagnostic Research and Reference Laboratory (DDRRL) during 2009 to 2015 after ethical approval by institutional review board (Ref no. IRB-459/DUHS/-14). Convenient non probability sampling method was used. All the cases of preneoplastic & neoplastic lesions of small and large intestine including biopsies as well as resection specimen received at DDRRL during the period of seven years were reviewed. The variables included registration number, age, gender, site of the lesion & diagnosis. Cases with inadequate data of the mentioned variables were excluded. The data obtained were entered and subjected to descriptive statistical analysis using SPSS version 22. Furthermore, the association of diagnosis was seen with various other variables including, gender & site of the lesion using Chi square test. To determine the association of age of the patient with diagnosis, patient’s age was divided into four groups. Group-I included patients in age between 15 to 35 years, group II = 36 to 55 years, group III = 56 to 75 years & group IV = 76 to 95 years of age. A p-value of < 0.05 was considered as significant.

## RESULTS

A total of 33 cases were diagnosed as premalignant lesions of intestine consisting of adenomatous polyp (AP) = 39.4% (n=13) [(males = 6.9% (n=10), females = 23% (n=3)], dysplasia = 36.4% (n=12) [males = 50% (n=6), females = 50% (n=6)] and adenoma 24.2% (n=8) cases [(males = 87.5% (n=7), females = 12.5%(n=1)). Insignificant association of premalignant lesions was seen with age, gender & site of the lesion (p = 0.073), (p = 0.155), (p = 0.654) respectively. Moreover, we also identified 15 cases of ulcerative colitis.

From the total of 453 cases diagnosed as malignant lesions of intestine, a total of 93.3% (n=423) cases were of adenocarcinoma, 2.8% (n= 13) cases were of squamous cell carcinoma (SCC), 2.4% (n=11) of metastatic adenocarcinoma, 0.9% (n=4) of neuroendocrine tumors and 0.4% (n=2) cases of lymphomas. Out of the total 423 cases of adenocarcinoma, Grade-I were 14.2% (n=64), Grade-II were 57.6% (n=260) and Grade-III were 22% (n=99) cases. Out of the total 13 cases of SCC, Grade-I were 0.4% (n=2), Grade-II were 1.6% (n=7) and Grade-III were 0.9% (n=4) cases.

**Fig.1:**
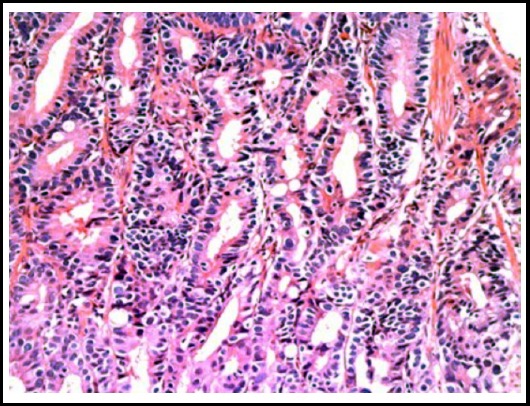
Colorectal adenocarcinoma Grade-II (H&E stain 20X).

A significant association was seen between site of the tumor and diagnosis, rectum was the commonest site for adenocarcinomas (p=0.001). We also noted a significant association between age group & diagnosis, where moderately differentiated adenocarcinoma was predominantly present in young age (age group II) (p=0.001). Although statistically we found insignificant association between gender and malignant lesions, they were predominantly seen in males as compared to females (p = 0.419) (Table I and II) (Fig.1).

**Table-I:** Gender distribution of malignant lesions.

Diagnosis	Gender		Total	p-value=0.428

	Male	Female		
Grade-I adenocarcinoma	6.25% (n=40)	37.5% (n=24)	64	
Grade-II adenocarcinoma	61.9% (n=161)	38% (n=99)	260	
Grade-III adenocarcinoma	63.6% (n=63)	36.3% (n=36)	99	
Metastatic adenocarcinoma	36.3% (n=4)	63.6%(n=7)	11	
Grade-I squamous cell Ca	50% (n=1)	50% (n=1)	2	
Grade-II squamous cell Ca	42.8% (n=3)	57.1% (n=4)	7	
Grade-III squamous cell Ca	50% (n=2)	50% (n=2)	4	
Neuroendocrine tumor	100% (n=4)	0	4	
Lymphoma	100% (n=2)	0	2	

* Pearson chi square, (level of significance at < 0.05), ** Ca = carcinoma.

**Table-II:** Age distribution of malignant lesions.

Diagnosis	Age groups				Total	p-value = 0.001

	Group I	Group II	Group III	Group IV		
Grade-I adenocarcinoma	22.3% (n=15)	35.8% (n=24)	32.8% (n=22)	4.4% (n=3)	67	
Grade-II adenocarcinoma	25.7% (n=67)	44.2% (n=115)	2.6% (n=7)	2.3% (n=6)	260	
Grade-III adenocarcinoma	50% (n=50)	30.3% (n=30)	13.1% (n=13)	6% (n=6)	99	
Metastatic adenocarcinoma	27.2% (n=3)	36.3% (n=4)	36.3% (n=4)	0	11	
Grade-I squamous cell Ca	0	50% (n=1)	0	50% (n=1)	2	
Grade-II squamous cell Ca	28.5%(n=2)	42.8% (n=3)	28.5% (n=2)	0	7	
Grade-III squamous cell Ca	0	75% (n=3)	25% (n=1)	0	4	
Neuroendocrine tumor	0	25% (n=1)	50% (n=2)	25% (n=1)	4	
Lymphoma	0	100% (n=2)	0	0	2	

* Pearson chi square, (level of significance at < 0.05).

## DISCUSSION

The incidence of colorectal cancer is on rise both in Western and Asian population.^1^ Worldwide, colorectal cancer ranks to be the 3^rd^ most common cancer whereas 2^nd^ in mortality wise.^1^ A wide variation for about 10 folds is observed in its incidence & 4-6 folds in its mortality rates across the globe.^4^ A rapidly rising trend is still seen in low and middle income countries including Pakistan whereas a declining or stabilizing pattern is noted in high income countries where their human development index (HDI) is highest.^4^ This disparity possibly can be related to their highest HDI where early detection, preventive measures and perioperative care is more stable. Majority of colorectal cancer develops from premalignant lesions. The most frequent preneoplastic lesion of intestine is adenoma, which is more common in large intestine as compared to small intestine.^5^ Studies in the past have recorded South Asian countries including Karachi to be in the low adenoma/adenomatous polyp prevalent area as compared to the Western and European countries.^6^ However, now a changing trend is being observed. Some Asian countries like Thailand, Korea and China show similar adenomatous polyp rate as in American and European countries.^7^,^8^ In our study, we found adenomatous polyp to be the most frequent preneoplastic lesion (39.3%). This is contrary to the study reported from Karachi in 2005 where they noted the frequency of adenomatous polyp was 12.7%.^6^ However, recent study from Mayo Clinic, US and India reported adenomatous polyp detection rate (ADR) of 31% and 41% respectively.^9^,^10^ A noticeable difference could be seen in one decade which shows a rising trend in the frequency of adenomatous polyp in South Asian population along with the Western countries. These differences might be due to increased westernized life style in our population including high fat diet and obesity. However, we found the frequency of adenomatous polyp was much lower than colorectal cancer in our series which is similar to another study done in our population.^11^ However, a reverse trend is observed from the countries where colon cancer screening programs are conducted.^6^ In Pakistan, cancer epidemiology is being widely studied by regional cancer registry centers, but, we are still deficient in having cancer registry at national level. Shaukhat Khanum Hospital reported colorectal cancer to be the 2^nd^ most common cancer for the year 2017.^12^ Whereas, PCR for the year 2016 ranked colorectal cancer as 4^th^ most common cancer.^13^ However, Karachi shows lower incidence of colorectal cancer where meat is not a regular diet due to unaffordability of Karachilites as compared to other cities of Pakistan.^14^ KCR stated a low but rising incidence of colorectal cancer in Pakistan especially in young men & advanced stages of the cancer.^15^ Our findings also revealed an estimated mean age of 46 years with male predominance (62.7%) for colorectal cancer which is in accordance with other regional studies.^16^ For instance, Ahmad et al. reported mean age of 40.92±14.73 years where majority (60%) were males and 40% were females.^16^ Similarly, Amini et al. also reaffirmed the general trend in our population of colorectal cancer presenting with majority of males at young age.^17^ In contrast, past data from developed countries reported higher incidence of CRC in sixth decade of life with rarity in early ages.^18^ Potential explanation for this difference might be presence of younger proportion of population which increases subject “at risk” & may be responsible for large number of cases. In our series, we observed moderately differentiated adenocarcinoma as the commonest lesion of intestine which is comparable with the study done by Gul A et al.^19^ Our study is also in agreement with other studies which observed rectum as the commonest site of intestinal cancer.^16^,^20^

With regards to small intestinal malignancies, it is rare, constituting only 20% of the gut tumors.^7^ Hence, its epidemiology is not well known. However, few studies have indicated low prevalence in Asia than West.^7^ Although the cause is unknown, it might be due to improved detection of the disease. Our series noted 12% of small intestinal cancers and duodenum being the most common site which is in accordance with other studies which observed duodenum being the most frequent (55-82%) followed by jejunum (11-25%) & ileum (7-17%).^21^ Several predisposing conditions like Crohns disease, celiac disease, adenomas and familial adenomatous polyposis contribute as risk factors for small intestinal cancers. Several behavioral risk factors could also be shared with colorectal cancer including lack of physical activity, high fat diet and smoking.

## CONCLUSION

Colorectal carcinoma is on rise in Pakistan, predominantly in young males, and rectum being the commonest site. In our study, all the lesions showed male predominance with adenomatous polyp as the commonest premalignant lesion & Grade-II adenocarcinoma the most common malignancy of intestine.

### Recommendations

Data pertaining preneoplastic lesions of intestine and small intestinal lesions is very scarce which needs to be investigated. As the illustrated data so far from Pakistan is from regional registries, there is a dire need to accumulate these data & establish a national level cancer registry from where the statistics can be referred.

### Authors’ Contribution:

**AS:** Conception of idea and manuscript drafting. Corresponding author and therefore take responsibility and accountability of the data/work presented herein.

**MAQ:** Manuscript drafting, proof reading and data analyses.

**AS and SA:** Involved in data collection.

**AS:** Contributed in manuscript writing.

**TM:** Did critical corrections.
